# The MAPK signaling pathway mediates the GPR91-dependent release of VEGF from RGC-5 cells

**DOI:** 10.3892/ijmm.2015.2195

**Published:** 2015-04-23

**Authors:** JIANYAN HU, TINGTING LI, SHANSHAN DU, YONGDONG CHEN, SHUAI WANG, FEN XIONG, QIANG WU

**Affiliations:** 1 Department of Ophthalmology, Shanghai Jiao Tong University Affiliated Sixth People’s Hospital, Shanghai 200233, P.R. China; 2 Shanghai Key Laboratory of Diabetes Mellitus, Shanghai 200233, P.R. China

**Keywords:** small hairpin RNA, G-protein-coupled receptor 91, vascular endothelial growth factor, mitogen-activated protein kinase signaling pathway, cyclooxygenase-2, prostaglandin E_2_, diabetic retinopathy

## Abstract

Vascular endothelial growth factor (VEGF) is one of the major regulatory molecules in diabetic retinopathy (DR). In our previous study, we demonstrated that succinate levels were elevated in the retinas of diabetic rats and that the knockdown of the succinate receptor, G-protein-coupled receptor 91 (GPR91), inhibited the release of VEGF and attenuated retinal vascular disorder in the early stages of DR. In the present study, we examined the signaling pathways involved in the GPR91-dependent release of VEGF in the retinal ganglion cell line, RGC-5. The cells were infected with a lentiviral small hairpin RNA (shRNA) expression vector targeting GPR91 (LV.shGPR91). Immunofluorescence staining revealed that GPR91 was predominantly localized in the cell bodies of the RGC-5 cells. RT-qPCR, western blot analysis and ELISA indicated that succinate exposure upregulated VEGF expression, activated the extracellular signal-regulated protein kinase (ERK)1/2, c-Jun N-terminal kinase (JNK) and p38 mitogen-activated protein kinase (MAPK) signaling pathways and led to the release of cyclooxygenase-2 (COX-2) and prostaglandin E_2_ (PGE_2_). The knockdown of GPR91 inhibited ERK1/2 and JNK activity, but did not inhibit the activation of the p38 MAPK pathway. The increase in COX-2 expression and the release of PGE_2_ were inhibited by transduction with LV.shGPR91 and ERK1/2, JNK and COX-2 inhibitors. The expression and release of VEGF showed similar results. Cell Counting Kit-8 (CCK-8) assays revealed that the shRNA-mediated knockdown of GPR91 decreased the proliferation of RF/6A cells cultured in succinate-conditioned medium. Our data suggest that GPR91 modulates the succinate-induced release of VEGF through the MAPK/COX-2/PGE_2_ signaling pathway.

## Introduction

Diabetic retinopathy (DR) is a major complication of diabetes mellitus and is one of the leading causes of acquired blindness. As the prevalence of DR has progressively increased worldwide, the management of DR has become a major goal of ophthalmic research (1). However, the fundamental cause of DR has not yet been fully elucidated. Although laser therapy has been shown to prevent partial visual loss to a certain extent, the current available treatments for DR are far from satisfactory; thus, the discovery of a specific therapeutic target for DR is required.

Vascular endothelial growth factor (VEGF) has long been recognized as the principal mediator of the process of DR and has been amply investigated (2,3). VEGF is a major cytokine that causes vascular leakage and induces endothelial cell division, proliferation, migration and capillary formation, leading to angiogenesis (4,5). Extensive evidence has indicated that VEGF expression is involved in the clinical association between retinal ischemia and retinal microvascular leakage (6,7). Sapieha *et al* (8) observed that G-protein-coupled receptor 91 (GPR91) was associated with neovascularization in ischemic retinopathy. GPR91, also known as succinate receptor 1 (SUCNR1), was first identified in 2004 as a specific receptor for succinate (9). GPR91 is highly expressed in a variety of highly vascularized organs, including the retina (8,10). GPR91 has been demonstrated to play critical roles in the pathogenesis of diabetic neuropathy, hypertension, heart stress and liver damage (11–14). In our previous study, we reported an accumulation of succinate during the early stages of DR, which activated GPR91, inducing the release of VEGF and attenuating retinal vascular dysfunction (10). Clinical research has detected high levels of succinate in patients with proliferative diabetic retinopathy (PDR) (15). Therefore, succinate-GPR91 signaling may play a vital role in the development and progression of DR. In another previous study of ours, we also demonstrated the role of GPR91 in the high-glucose-induced release of VEGF (16). We also previously indicated that extracellular signal-regulated kinase (ERK)1/2 has an effect on the succinate-GPR91 pathway *in vivo* (10). However, the underlying mechanisms of action of the mitogen-activated protein kinase (MAPK) signaling pathway have not yet been fully elucidated.

As has been reported in studies on diabetic nephropathy, the succinate-GPR91 pathway mediates the upregulation of MAPK, cyclooxygenase-2 (COX-2) and prostaglandin E_2_ (PGE_2_) (17,18). Previous studies have demonstrated that G-protein-coupled receptors regulate the activation of the MAPK signaling pathway (19,20). The MAPK signaling pathway influences a broad array of cellular processes, including embryogenesis, proliferation, differentiation and apoptosis by regulating the cell cycle engine and other cell proliferation-related proteins (21,22). There are at least three MAPK families: i) ERK1/2; ii) c-Jun N-terminal kinase (JNK); and iii) p38 MAPK or the stress/cytokine activated kinase. The results of recent studies support the hypothesis that the MAPK signaling pathway affects the pathogenesis of DR (23–25). Moreover, studies have demonstrated that elevated levels of COX-2 and COX-2-induced PGE_2_ in various tissues are associated with the upregulation of VEGF (26–28). Additionally, VEGF expression has been shown to be inhibited by COX-2 inhibitors in the diabetic retina (29). In the present study, we examined the hypothesis that the MAPK (including ERK1/2, JNK and p38 MAPK) signaling pathway mediates the GPR91-dependent release of VEGF from retinal ganglion cells (RGCs).

## Materials and methods

### Cell lines culture and treatment

The retinal ganglion cell line, RGC-5, from the American Type Culture Collection (ATCC, Manassas, VA, USA) was cultured in Dulbecco’s modified Eagle’s medium (DMEM; HyClone Laboratories, Red Bank, NJ, USA) containing 10% fetal bovine serum (FBS; Hyclone Laboratories) and 1% penicillin/streptomycin mixture (HyClone Laboratories) at 37°C in a humidified atmosphere of 5% of CO_2_. The rhesus retinal vascular endothelial cell line, RF/6A (from the Cell Bank of the Chinese Academy of Sciences, Shanghai, China), was cultured in RPMI-1640 medium (Gibco, Grand Island, NY, USA) containing 10% FBS and 1% penicillin/streptomycin at 37°C in a humidified atmosphere of 5% CO_2_. The culture medium was replaced with fresh medium every other day, and the cells were passaged every 2–3 days. The RGC-5 cells were incubated with succinate with or without pre-treatment with 10 *µ*M of the ERK1/2 inhibitor, U0126 (Calbiochem, Gibbstown, NJ, USA), 10 *µ*M of the JNK inhibitor, SP600125 (Calbiochem), or 50 *µ*M of the COX-2 inhibitor, NS-398 (Cayman Chemical Co., Ann Arbor, MI, USA).

### GPR91 small hairpin RNA (shRNA) design, lentivirus packaging and gene transduction

The complete sequence of the rat GPR91 sequence was obtained from NCBI (GenBank Accession Number: NM_001001518) and a suitable shRNA target sequence (AACCCTAAATACAGTCTCATT) was selected. The shRNA oligonucleotide fragments were annealed to form the target double-stranded DNA, followed by digestion with *Age*I and *Eco*RI and ligation with T4 DNA ligase to insert the shRNA into the lentivirus expression vector, pGCSIL-GFP (Shanghai Genechem Co., Ltd., Shanghai, China). The plasmids were then transformed into competent *E. coli* DH5α cells and screened on plates containing antibiotics to produce the recombinant pGCSIL-GFP-GPR91-shRNA vector and the control lentivirus containing a non-silencing shRNA (LV.shScrambled). The lentivirus containing shRNA targeting GPR91 was produced by the plasmid co-transfection of 293T cells according to previously described methods (16).

### Immunofluorescence staining

The RGC-5 cells grown on cover-slips were fixed in 4% paraformaldehyde for 30 min at room temperature, followed by permeabilization with 0.1% Triton X-100 for 20 min at 4°C. The cells were blocked in 5% BSA for 1 h at room temperature, followed by overnight incubation at 4°C with the primary antibodies: rabbit anti-rat GPR91 polyclonal antibody (1:200; NBP1-00861; Novus Biologicals LLC, Littleton, CO, USA), rabbit anti-rat p-ERK1/2 monoclonal antibody (1:200; #4370), rabbit anti-rat p-JNK monoclonal antibody (1:200; #4668) and rabbit anti-rat p-p38 monoclonal antibody (1:200; #4511; Cell Signaling Technology, Boston, MA, USA). The cells were incubated with fluorescein isothiocyanate (FITC)-conjugated secondary antibodies (1:200; A21206; Invitrogen, Carlsbad, CA, USA) for 1 h at room temperature.

### Reverse transcription-quantitative PCR (RT-qPCR)

Total RNA was extracted using TRIzol reagent (Invitrogen). The purity of the RNA (A260/A280 >1.8) was examined using a spectrophotometer, and the integrity of the RNA was confirmed by visualization of the 28S and 18S bands (2:1) on 1% agarose gels. The RNA (1 *µ*g) was used to synthesize cDNA using the SuperScript First-Strand Synthesis kit (Fermentas, Pittsburgh, PA, USA) following the manufacturer’s instructions.

Quantitative PCR was then performed using the SYBR-Green qPCR Super Mixture (Takara, Shiga, Japan) and an ABI Prism 7500 Sequence Detection system (Life Technologies, Grand Island, NY, USA). The following primer sequences were used: rat VEGF (forward, 5′-AAAGCC AGCACATAGGAGAG-3′ and reverse, 5′-AGGATTTAAACCGGGATTTC-3′); COX-2 (forward, 5′-TACAACAACTCCATCCTCCTTG-3′ and reverse, 5′-TTCATCTCTCTGCTCTGGTCAA-3′) and rat β-actin (forward: 5′-CACCCGCGAGTACAACCTTC-3′ and reverse, 5′-CCCATACCCACCATCACACC-3′). The amplification conditions were as follows: 95°C for 10 min followed by 40 cycles of 95°C for 15 sec, 60°C for 60 sec and 72°C for 60 sec. The specificity of the detected signals was confirmed by a dissociation curve, which consisted of a single peak. All samples were run in triplicate in each experiment. Data were analyzed using the 2^−ΔΔCT^ method.

### Western blot analysis

The harvested cells were lysed in lysis buffer on ice and the cell extracts were clarified by centrifugation (at 9,500 × g). The protein concentrations were determined by spectrophotometry using the Pierce™ BCA Protein Assay kit (Thermo Fisher Scientific, Rockford, IL, USA). Aliquots containing 30 *µ*g of protein were separated by SDS-polyacrylamide gel electrophoresis on a 10% gel, and the separated proteins were blotted onto polyvinylidene difluoride membranes in a wet transfer unit (Bio-Rad Laboratories, Hercules, CA, USA). After blocking with 5% non-fat dry milk at room temperature for 1 h, the membranes were incubated overnight at 4°C with following primary antibodies: rabbit anti-rat GPR91 polyclonal antibody (1:1,000), rabbit anti-rat p-ERK1/2 monoclonal antibody (1:3,000), rabbit anti-rat ERK1/2 monoclonal antibody (1:3,000; #4695), rabbit anti-rat p-JNK monoclonal antibody (1:1,000), rabbit anti-rat JNK monoclonal antibody (1:1,000; #9252), rabbit anti-rat p-p38 monoclonal antibody (1:1,000), rabbit anti-rat p38 monoclonal antibody (1:1,000; #9212), goat anti-rat COX-2 polyclonal antibody (1:200; sc-1745; Santa Cruz Biotechnology, Dallas, TX, USA) and mouse anti-rat β-actin monoclonal antibody (1:5,000; A5441; Sigma-Aldrich, St. Louis, MO, USA). The bands were visualized using an enhanced ECL detection kit (Pierce Biotechnology, Rockford, IL, USA).

### Enzyme-linked immunosorbent assay (ELISA)

The supernatant medium was collected from non-transduced, LV.shScrambled-transduced or LV.shGPR91-transduced cells for ELISA. The levels of PGE_2_ and VEGF in the culture supernatants were detected separately by PGE_2_ and VEGF enzyme-linked immunoassay kits (R&D Systems, Minneapolis, MN, USA), following the manufacturer’s instructions. Each sample was tested in triplicate and the average result was reported.

### Cell Counting Kit-8 assay for the measurement of RF/6A cell proliferation using conditional medium from succinate-stimulated RGC-5 cells

The conditioned medium was collected from the succinate-stimulated RGC-5 cells, with or without shGPR91 transduction and pre-treatment with MAPK inhibitors. It was filtered through a 0.22-mm filter in a manner similar to that outlined in our previous study (16). The RF/6A cells were seeded in 96-well plates at a density of 5×10^3^ cells/100 *µ*l. The plate was pre-incubated overnight in a humidified incubator (37°C, 5% CO_2_). The cells were then exposed to the conditioned medium for 24 h in the same incubator and 10 *µ*l Cell Counting Kit-8 solution (Dojindo Laboratories, Kumamoto, Japan) was added to each well of the plate. Following incubation at 37°C for 4 h, the plates were analyzed using an ELISA reader at 450 nm.

### Statistical analysis

All data are presented as the means ± standard deviation (SD). Data were analyzed using SPSS 16.0 software (version 17.0; SPSS, Inc., Chicago, IL, USA). The differences between multiple groups were assessed by a one-way analysis of variance (ANOVA) followed by Student-Newman-Keuls (SNK) comparisons. A value of P<0.05 was considered to indicate a statistically significant difference.

## Results

### GPR91 is located in RGC-5 cells

**Figure 1:**
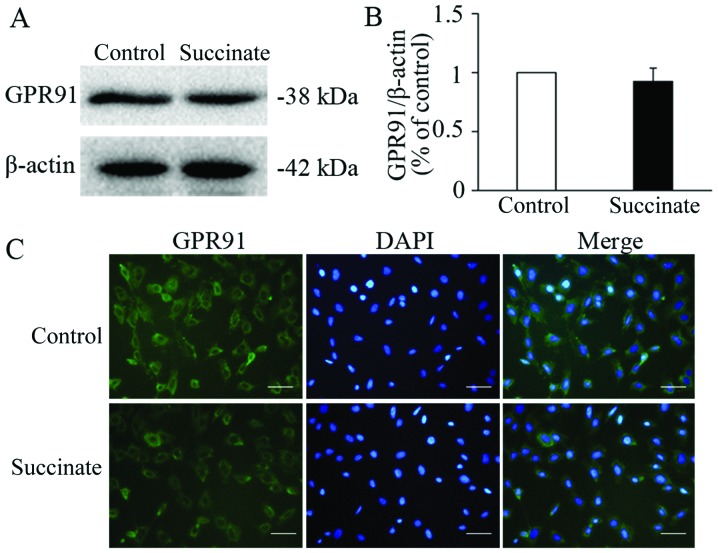
G-protein-coupled receptor 91 (GPR91) is expressed in RGC-5 cells. (A) Western blot analysis of GPR91 protein expression in RGC-5 cells treated with 50 *µ*M succinate for 24 h. (B) Quantitative analysis of the band density of GPR91/β-actin. Each column denotes the mean ± SD (n=3). (C) Immunofluorescence images showing the location of GPR91 in the cytoplasm of RGC-5 cells treated with succinate for 24 h. Scale bar, 50 *µ*m.

First of all, we observed the expression and location of the GPR91 receptor in the RGC-5 cells. Western blot analysis did not reveal any significant differences in GPR91 expression when the RGC-5 cells were incubated with 50 *µ*M succinate for 24 h (Fig. 1A and B). GPR91 was located in the cell bodies of the RGC-5 cells incubated with or without succinate (Fig. 1C).

### GPR91 modulates the succinate-induced increase in ERK1/2 and JNK signaling in RGC-5 cells

**Figure 2:**
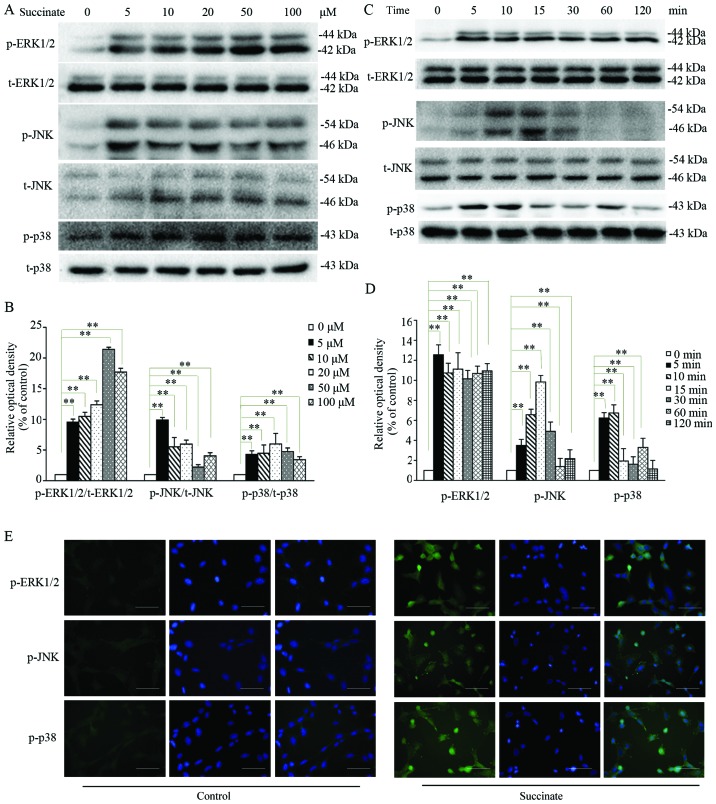
Succinate-induced activation of ERK1/2, JNK and p38 MAPK signaling pathways in RGC-5 cells. (A) Western blot analysis of ERK1/2, JNK and p38 MAPK phosphorylation in RGC-5 cells incubated with various concentrations of succinate for 10 min. (B) Quantitative analysis of the band density. Each column represents the mean ± SD (n=3). (C) Western blot analysis of ERK1/2, JNK and p38 MAPK phosphorylation in RGC-5 cells treated with 10 *µ*M of succinate for different periods of time. (D) Quantitative analysis of the band density. Each column represents the mean ± SD (n=3). (E) Immunofluorescence images showing ERK1/2, JNK and p38 MAPK in the cytoplasm of RGC-5 cells treated with 10 *µ*M succinate for 10 min. ^**^P<0.01 vs. the untreated control. Scale bar, 50 *µ*m.

Next, we attempted to clarify the signaling mechanisms of action of GPR91 in the succinate-treated RGC-5 cells. As shown in Fig. 2A–D, the results revealed that treatment with succinate activated ERK1/2, JNK and p38 MAPK signaling. The cells incubated with various concentrations of succinate for 10 min exhibited a dose-dependent increase in MAPK signaling, and a time-dependent trend was also observed when the cells were incubated with 10 *µ*M succinate. Succinate induced a >2-fold increase in the p-ERK1/2, p-JNK and p-p38 MAPK levels in the RGC-5 cells (Fig. 2A–D). The results from immunofluorescence staining indicated that p-ERK1/2, p-JNK and p-p38 became notably more visible in the cytoplasm following incubation with 10 *µ*M succinate for 10 min (Fig. 2E).

**Figure 3:**
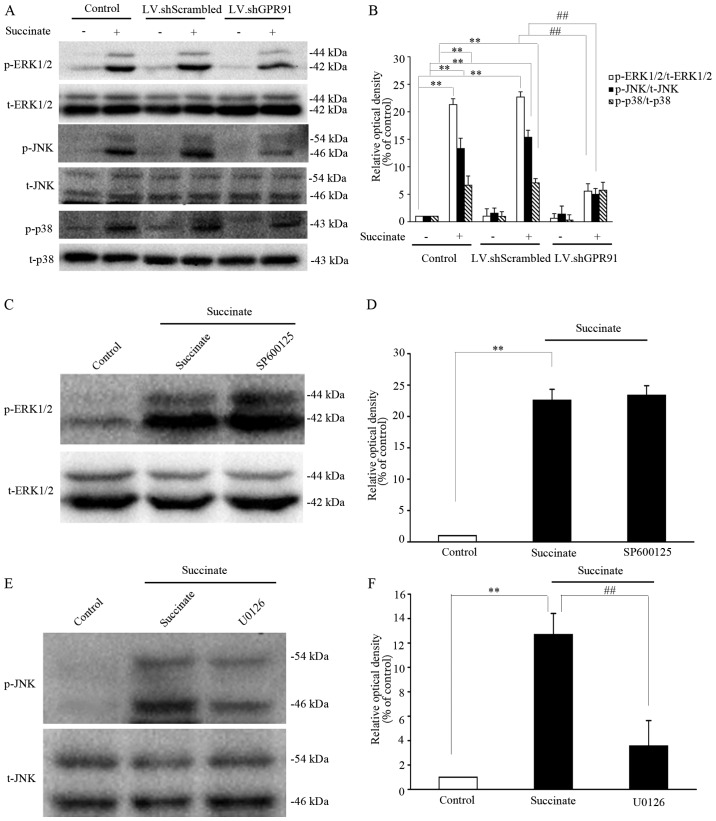
G-protein-coupled receptor 91 (GPR91) modulates succinate-induced ERK1/2 and JNK signaling in RGC-5 cells. (A) Changes in ERK1/2, JNK and p38 MAPK phosphorylation (determined by western blot analysis) in RGC-5 cells transduced with LV.shScrambled or LV. shGPR91. (B) Quantitative analysis of the band density. Each column represents the mean ± SD (n=3). (C) Effect of the JNK inhibitor, SP600125, on ERK1/2 phosphorylation in RGC-5 cells treated with succinate. (D) Quantitative analysis of the band density. Each column represents the mean ± SD (n=3). (E) Effect of the ERK1/2 inhibitor, U0126, on JNK phosphorylation in RGC-5 cells treated with succinate. (F) Quantitative analysis of the band density. Each column represents the mean ± SD (n=3). ^**^P<0.01 vs. the untreated control. ^##^P<0.01 vs. LV.shScrambled-transduced cells.

In order to assess whether succinate modulates the activation of the MAPK signaling pathway through its receptor, GPR91, we incubated the RGC-5 cells with succinate (50 *µ*M to activate ERK1/2, 5 *µ*M for JNK and 20 *µ*M for p38 MAPK) for 10 min. The increase in the phosphorylation levels of ERK1/2 and JNK was significantly blocked by GPR91 shRNA (P<0.01); however, p38 MAPK phosphorylation was not affected (P>0.05). These results indicate that GPR91 mediates the activation of ERK1/2 and JNK signaling in RGC-5 cells (Fig. 3A and B).

Treatment with MAPK signaling inhibitors also revealed that the treatment of the RGC-5 cells with succinate increased JNK activity in an ERK1/2-dependent manner. ERK1/2 phosphorylation was not significantly altered when the RGC-5 cells were pre-treated with 10 *µ*M SP600125 (JNK inhibitor, P>0.05; Fig. 3C and D), whereas JNK phosphorylation decreased significantly when the cells were pre-treated with U0126 (ERK1/2 inhibitor, P<0.01; Fig. 3E and F).

### GPR91 modulates the activation of the ERK1/2 (JNK)/COX-2/PGE_2_ pathway in RGC-5 cells

**Figure 4:**
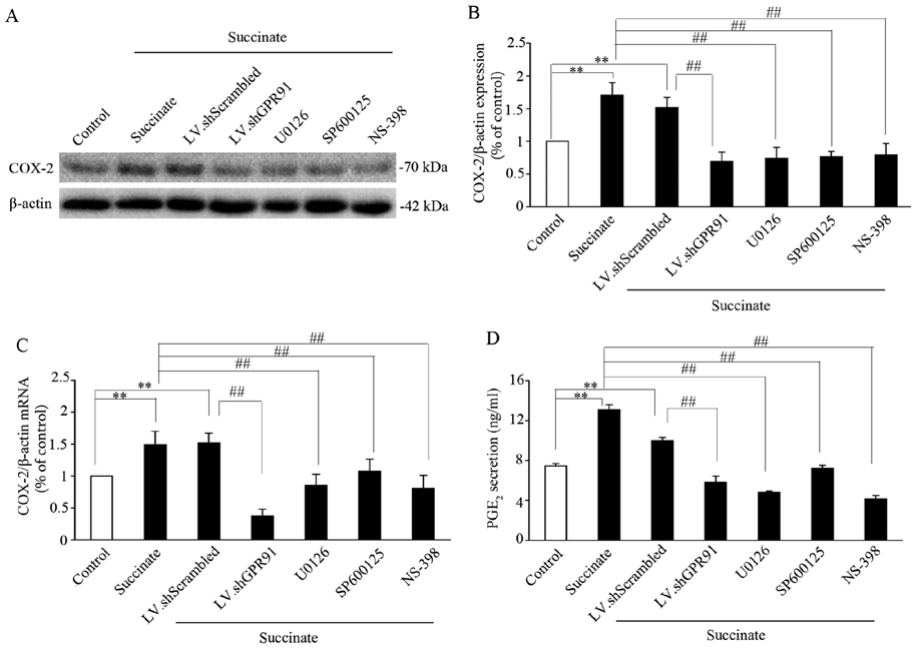
G-protein-coupled receptor 91 (GPR91) modulates the succinate-induced activation of the ERK1/2(JNK)/COX-2/PGE_2_ pathway in RGC-5 cells. (A) Western blot analysis of COX-2 protein expression in RGC-5 cells treated with succinate for 24 h. The cells were transduced with LV.shGPR91 or pre-treated with U0126 (ERK1/2 inhibitor), SP600125 (JNK inhibitor) or NS-398 (COX-2 inhibitor). (B) Quantitative analysis of the band density of COX-2/β-actin. (C) Changes in COX-2 mRNA levels (determined by RT-qPCR) in RGC-5 cells from each group. (D) Changes in PGE_2_ release (determined by ELISA) by RGC-5 cells from each group. Each column represents the mean ± SD (n=3). ^**^P<0.01 vs. the untreated control. ^##^P<0.01 vs. succinate-treated cells.

To examine whether COX-2 expression remains elevated following incubation with succinate, we exposed the RGC-5 cells to 50 *µ*M succinate for 24 h. COX-2 expression increased significantly (P<0.01; Fig. 4A and C). PGE_2_ levels were also measured as the production of PGE_2_ denotes COX-2 activity; the PGE_2_ levels were also markedly increased in the succinate-stimulated RGC-5 cells (P<0.01; Fig. 4D). However, the upregulation of COX-2 expression and activity were markedly decreased following the knockdown of GPR91 (P<0.01; Fig. 4A and D). Furthermore, pre-treatment with 10 *µ*M U0126 (ERK1/2 inhibitor), 10 *µ*M SP600125 (JNK inhibitor) or 50 *µ*M NS-398 (COX-2 inhibitor) significantly blocked the upregulation of COX-2 and PGE_2_ expression in the RGC-5 cells (P<0.01). These findings indicate that the ERK1/2 and JNK pathway is upstream of the COX-2 and PGE_2_ pathway.

### GPR91 modulates the succinate-induced release of VEGF through the ERK1/2(JNK)/COX-2/PGE_2_ pathway in RGC-5 cells

**Figure 5:**
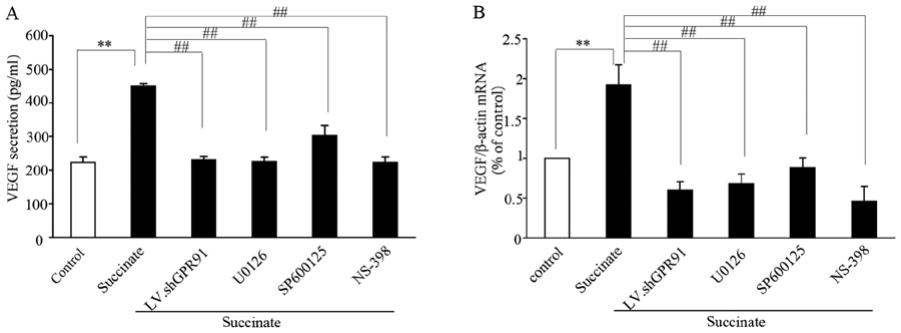
G-protein-coupled receptor 91 (GPR91) modulates the succinate-induced release of vascular endothelial growth factor (VEGF) through the ERK1/2 (JNK)/COX-2/PGE_2_ pathway in RGC-5 cells. (A) Changes in VEGF release (determined by ELISA) in RGC-5 cells treated with succinate for 24 h. The cells were transduced with LV.shGPR91 or pre-treated with U0126 (ERK1/2 inhibitor), SP600125 (JNK inhibitor) or NS-398 (COX-2 inhibitor). (B) Changes in VEGF mRNA levels (determined by RT-qPCR) in RGC-5 cells from each group. Each column represents the mean ± SD (n=3). ^**^P<0.01 vs. the untreated control. ^##^P<0.01 vs. succinate-treated cells.

We then investigated the association between GPR91 and the succinate-induced increase in VEGF expression. The expression and secretion of VEGF by these cells was significantly downregulated following transduction with LV.shGPR91 or pre-treatment with 10 *µ*M U0126 (ERK1/2 inhibitor), 10 *µ*M SP600125 (JNK inhibitor) or 50 *µ*M NS-398 (COX-2 inhibitor), and these differences were statistically significant (P<0.01; Fig. 5A and B).

### GPR91 modulates the proliferation of rhesus vascular endothelial RF/6A cells treated with conditional medium from succinate-stimulated RGC-5 cells

**Figure 6:**
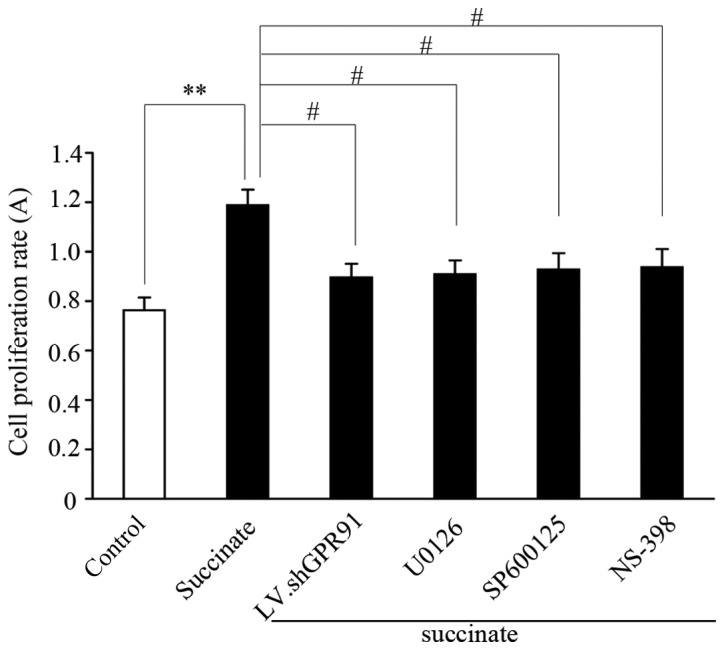
G-protein-coupled receptor 91 (GPR91) modulates the proliferation of RF/6A cells treated with conditioned medium from RGC-5 cells. Changes in cell proliferation rates in RF/6A cells treated with conditioned medium for 24 h. The RGC-5 cells were stimulated with succinate, with or without LV.shGPR91 transduction, or U0126, SP600125 and NS-398 pre-treatment. Each column represents the mean ± SD (n=3). ^**^P<0.01 vs. untreated control. #P<0.05 vs. succinate-stimulated cells.

We also used CCK-8 assays to observe the proliferation of RF/6A cells following incubation with succinate-conditioned medium for 24 h. The proliferation of the RF/6A cells markedly increased upon incubation with the conditioned medium (P<0.01). Following incubation with the medium from the LV.shGPR91-transduced RGC-5 cells, the U0126 pre-treated RGC-5 cells, the SP600125 pre-treated RGC-5 cells or the NS-398 pre-treated RGC-5 cells, the proliferation of the RF/6A cells markedly decreased (P<0.01; Fig. 6).

## Discussion

In a previous study of ours, we demonstrated that succinate was accumulated in the retinas of diabetic rats and activated its special receptor, GPR91, which is primarily expressed in the layer of ganglion neural cells. We also demonstrated that ERK1/2 mediated the effects of GPR91 during the early stages of DR (10). In the present study, we used succinate-stimulated RGCs to determine the effects of 3 major subfamilies of the MAPK signaling pathway (ERK1/2, JNK and p38 MAPK) on the regulatory mechanisms of GPR91. Our findings indicated that succinate stimulation led to several early biochemical events, including the activation of ERK1/2, JNK, p38 MAPK, COX-2 and PGE_2_, and an increase in VEGF expression. The ERK1/2, JNK and p38 MAPK sigaling pathways mediate a variety of downstream biological effects. ERK1/2 signaling is associated with oncogenesis (20), diabetic complications (30) and angiogenesis (31). JNK signaling is associated with cell apoptosis, transformation and oncogenesis and interacts with other cytokine-mediated pathways (32). In addition, p38 MAPK is considered to be involved in inflammation in a number of diseases (33–35). Although exogenous succinate activated ERK1/2, JNK and p38 MAPK, our results revealed that the phosphorylation of ERK1/2 and JNK was downregulated to a significant degree following the transduction of the RGC-5 cells with LV.shGPR91, but this was not true for p38 MAPK. These findings suggest that succinate enhances the GPR91-mediated activation of the ERK1/2 and JNK pathways in RGC-5 cells. We speculate that the findings regarding p38 may be due to the following reasons: first, crosstalk may exist that leads to interactions and influences among the ERK1/2, JNK and p38 MAPK signaling pathways. For example, JNK is a scaffold for JIP2, which has been demonstrated to be a member of the p38 MAPK family through binding to p38α and p38δ (22). Using our target RGC-5 cells, we also found that JNK activity was ERK1/2-dependent. Second, although p38 MAPK signaling is believed to play a role in the histopathology of DR (23,25), other metabolic products, such as adenosine triphosphate (ATP) intermediates may lead to the activation of p38 MAPK signaling. ATP has been reported to activate p38 MAPK signaling by binding the P2Y receptor (36,37). However, further research is required to determine the mechanisms underlying GPR91 activity.

In this study, succinate stimulation significantly increased the levels of COX-2 and PGE_2_ in the RGC-5 cells. As the COX-2 gene encodes a cytosolic protein that is upregulated during inflammation, an elevated COX-2 expression suggests that inflammation plays a critical role in the occurrence and development of DR (38). Previous studies have indicated that inflammatory cytokines are closely associated with retinal ischemia and the neovascularization characteristics of DR (39,40). Additionally, studies have suggested that the release and secretion of VEGF induced by cyclooxygenases is an important factor (27,28,41). All the above-mentioned results demonstrate that by mediating an inflammatory response, COX-2 may contribute to local ischemia and hypoxia in the retina and may thus induce the release of VEGF, ultimately leading to pathological angiogenesis. Our findings also demonstrated that the inhibition of COX-2 was effective in reducing succinate-induced VEGF expression, similar to PGE_2_ release. COX-2 activity results in the formation of 5 biologically active prostanoids (prostaglandins and thromboxanes) (42). Among these, PGE_2_, one of the most well-studied prostaglandins, is expressed in a number of tissues and has been implicated in the regulation of VEGF release (43–45). Zhong *et al* (46) found that the effect of insulin on mechanical strain-induced COX-2 expression was inhibited by the blockade of the ERK pathway. Chuang *et al* (47) demonstrated that a JNK signaling inhibitor significantly inhibited LPS-induced COX-2 expression. The results of our study also established that GPR91 shRNA, an ERK1/2 inhibitor, a JNK inhibitor and a COX-2 inhibitor all led to a marked decrease in the succinate-induced expression of COX-2 and PGE_2_, and the release of VEGF. Therefore, we hypothesized that the effect of GPR91 on VEGF secretion was at least partially regulated through ERK1/2(JNK)/COX-2/PGE_2_ in RGC-5 cells.

VEGF is a key factor in the occurrence and development of DR (2,3). The present study demonstrated that GPR91 was associated with VEGF overexpression in RGC-5 cells following succinate stimulation. We also found that the incubation of RF/6A cells with conditioned medium from succinate-stimulated RGC-5 cells led to a significant increase in cell proliferation. The proliferation of retinal endothelial cells leads to the sight threatening complications associated with DR (48). Under normal circumstances, the endothelium in these vascular beds is at rest. Under pathological conditions, endothelial cells begin to proliferate due to the overexpression of growth factors (48). It has been reported that VEGF is a potent mitogen for micro- and macrovascular endothelial cells derived from arteries, veins and lymphatics (49). Our results were similar to the those of the study by Li *et al* (50), who demonstrated that the proliferation of co-cultured choroidal endothelial cells was reduced by the downregulation of VEGF expression.

Collectively, the present study demonstrated the mechanisms of GPR91 regulation in RGC-5 cells following succinate stimulation. Using *in vitro* models, we demonstrate that GPR91 mediates VEGF secretion and endothelial cell proliferation, possibly by activating the ERK1/2 and JNK signaling pathways and then upregulating COX-2 and PGE_2_ expression. The data from this study may help to elucidate the mechanisms of GPR91-dependent signaling and may prove useful to the development of more effective therapies to prevent the development of DR.

## Abbreviations

ANOVA: analysis of variance
DR: diabetic retinopathy
SD: standard deviation
RGCs: retinal ganglion cells
FBS: fetal bovine serum
